# Work-related complaints and diseases of physical therapists – protocol for the establishment of a “Physical Therapist Cohort” (PTC) in Germany

**DOI:** 10.1186/1745-6673-8-34

**Published:** 2013-12-13

**Authors:** Maria Girbig, Stefanie Deckert, Christian Kopkow, Ute Latza, Madeleine Dulon, Albert Nienhaus, David Groneberg, Andreas Seidler

**Affiliations:** 1 Institute and Policlinic of Occupational and Social Medicine, Medical Faculty, Technical University Dresden, Fetscherstr. 74, D-01307 Dresden, Germany; 2 Federal Institute for Occupational Safety and Health, Nöldnerstr. 40/42, D-10317 Berlin, Germany; 3 Institution for Statutory Accident Insurance and Prevention in the Health and Welfare Services, Department of Occupational Health Research, Pappelallee 33-37, D-22089 Hamburg, Germany; 4 Institute of Occupational, Social and Environmental Medicine, Goethe-University, Theodor-Stern-Kai 7, D-60590 Frankfurt am Main, Germany

**Keywords:** Study protocol, Cohort study, Physical therapist, Occupational exposure, Occupational health, Occupational disease

## Abstract

**Background:**

Only few studies deal with the workload of physical therapists and the health consequences, although this occupational group is quite important for the health care system in many industrialized countries (e.g. ca. 136 000 people are currently employed as physical therapists in Germany). Therefore, the current state of knowledge of work-related diseases and disorders of physical therapists is insufficient. The aim of the "Physical Therapist Cohort" (PTC) study is to analyze the association between work-related exposures and diseases among physical therapists in Germany. This article describes the protocol of the baseline assessment of the PTC study.

**Methods/Design:**

A cross-sectional study will be conducted as baseline assessment and will include a representative random sample of approximately 300 physical therapists employed in Germany (exposure group), and a population-based comparison group (n = 300). The comparison group will comprise a sample of working aged (18–65 years) inhabitants of a German city. Variables of interest will be assessed using a questionnaire manual including questions regarding musculoskeletal, dermal, and infectious diseases and disorders as well as psychosocial exposures, diseases and disorders. In addition to subjective measures, a clinical examination will be used to objectify the questionnaire-based results (n = 50).

**Discussion:**

The study, which includes extensive data collection, provides a unique opportunity to study the prospective association of work-related exposures and associated complaints of physical therapists. Baseline results will give first clues with regard to whether and how prevalent main exposures of physiotherapeutic work and typical work areas of physical therapists are associated with the development of work-related diseases. Thereby, this baseline assessment provides the basis for further investigations to examine causal relationships in accordance with a longitudinal design.

## Background

Prompted by demographic change and the centrality of population-based medical (health-)care, numerous studies on work-related exposures and diseases among health care workers in Germany have been performed. The scientific interest in non-medical professionals has focused mainly on occupational groups such as caregivers and nurses [1-3]. Whereas workload of physical therapists, who currently represent about 136 000 employees in Germany [4] and are therefore an important occupational group of the health care system, have only been the subject of single national studies. The few existing articles focusing physical therapists published in ***Germany*** indicate that the physiotherapeutic work is associated with psychosocial [5,6] and dermal exposures [7] as well as diseases [8].

Given international studies musculoskeletal diseases, skin diseases, infections as well as mental complaints seem to be relevant for physical therapists. The consideration of ***international literature*** on the current state of research depicts the following facts:

### Musculoskeletal workload and diseases

Several international cross-sectional studies illustrate the relevance of musculoskeletal workload among physical therapists [9-12]. Lumbar spine, upper back, neck, shoulders, wrists/hands and knees were mentioned as main localizations of these complaints [9-15]. With regard to international investigations the following occupational diseases (defined officially in the German Ordinance on Occupational Diseases) [16] caused by physical impact could be relevant to physical therapists in Germany: Diseases of the tendon sheaths (No. 2101), disc-related diseases of the lumbar spine (No. 2108), osteoarthritis of the knee (No. 2112), and meniscus lesions (No. 2102).

Apparently, younger therapists (under 30 years) are especially affected by diseases of the musculoskeletal system in the first years after their qualification [9,11,12]. Nevertheless, results of longitudinal observations which could detect the potential consequences of long-term physical workload on the recurring complaints of physical therapists are missing. Some cross-sectional studies indicated exposures of physiotherapy work which seem to be relevant in this way, for example: high frequency of treatments [9,11,12], lifting and transferring of patients [13,17,18], bent and twisted treatment positions [10,11,17] as well as the application of manual techniques [12] are missing.

### Skin diseases

Masseurs, balneotherapists, and physical therapists employed in the field of medical rehabilitation and public welfare often suffer from work-related skin diseases. In this context, hand eczema is the most frequently reported disease and the appropriate occupational disease: “Severe or recurrent skin disease” (No. 5101) could be relevant [16]. Even if work-related skin diseases among healthcare/nursing and therapeutic professions are well documented, studies about dermal exposures are currently needed [19,20].

### Infectious diseases

Contact with patients’ open wounds (e.g. risk of hepatitis C, hepatitis B or HIV infection) or with infectious patients (e.g. tuberculosis and Methicillin-Resistant Staphylococcus aureus (MRSA)) is related to an increased risk of infection [21,22]. It remains unclear whether specific physiotherapeutic work areas and work tasks are associated with an increased risk of infection. But the occupational disease which picks infectious diseases in cases where the insured person worked in health care (No. 3101) as a central theme could be relevant [16].

### Mental complaints

The burnout syndrome is comparatively the most frequently studied mental outcome among physical therapists [23-25]. All in all, physical therapists tend to display a moderately increased prevalence of burnout syndrome.

So far, there is no study which has examined the all-encompassing wide spectrum of exposures and diseases associated with physiotherapeutic work. Evidence predominantly stems from cross-sectional designs and for this reason the potential etiologic role of physiotherapeutic work on specific diseases remains unclear. Furthermore it remains unclear whether the previous international research results are applicable to physical therapists currently working in Germany [5,6,8,26].

## Objective

The aim of the present study is the in-depth analysis of professional exposures, as well as health-related outcomes, of physical therapists in Germany. The baseline examination primarily focuses on musculoskeletal and dermal complaints and diseases as well as infections as outcomes. Mental complaints are considered only marginal at this time (by information of self-reported medical diagnosis). In accordance with the described purposes, the following research questions are underlying the study presented here.

### Primary research question

Is the work of physical therapists associated with a higher prevalence of musculoskeletal complaints and diseases compared to the general working population?

### Secondary research questions

Is the work of physical therapists, in comparison to the general working population, associated with a higher prevalence of:

I. Complaints and diseases of the skin

II. Infectious agents and infectious diseases, or

III. Mental complaints and diseases?

Moreover, the link between specific complaints of physical therapists in general and concrete physiotherapeutic work tasks (e.g. lifting/transferring dependent patients, working in awkward or cramped position) and work settings (e.g. outpatient vs. inpatient; pediatric clinic vs. orthopedics; employment vs. self-employment) will be determined.

## Methods/Design

### Study design and sampling frame

A cross-sectional design will be carried out as baseline assessment of the "Physical Therapist Cohort" (PTC) study. While physical therapists are considered to be exposed, working-aged (18 till 65 years) inhabitants of a German city (Dresden) will be included as external reference group.

### Sample size and power calculation

The primary research question refers to the prevalence of work-related musculoskeletal complaints and diseases. Low back pain is considered as particularly relevant for the occupational group of physical therapists.

The prevalence data of low back pain in the general German population (control group) was previously estimated within the “German Spine Study” (EPILIFT) [27] using the question, *“Have you been in medical treatment due to lower back pain during the last 12 months?”*. 24% of the male and 28% of the female German Spine Study participants reported requiring medical treatment (by a physician) for back pain in the previous year [27]. This may be a conservative estimation of prevalence compared to the planned study, because the analogous question used in this study includes in addition to the treatment by a physician, chiropractor and/or physical therapy treatments as well (*“Have you visited a physician, chiropractor or physical therapist due to lower back pain during the last 12 months?”*).

Sample size calculations were conducted using the WinPepi power analysis program [28]. The calculations were based on a 28% 12-month prevalence of medically treated lower back pain in the general population and the assumption that current study will estimate a prevalence ratio of at least 1.5 among physical therapists compared to the general population with a power of at least 80% (two-sided test). According to these assumptions, a minimum sample size of 182 persons is needed in each group. A response rate of more than 50% will be aspired. Based on the aimed response rate and the required sample size, 600 persons will be asked to participate in the survey for each group. Hopefully, this will result in the recruitment of approximately 300 physical therapists working in Germany, as well as a comparison group of also approximately 300 inhabitants of a German city (Dresden).

## Recruitment procedure

### Exposure group - physical therapists

The study base of the random sample of physical therapists will be selected from currently active professionals in Germany (n ≈ 136 000) [4]. Because physical therapists in Germany are not officially registered, a central recruitment is not possible. Thus an unbiased recruitment of the population of interest is difficult to realize. For this reason, two strategies of recruitment will be used to address approximately 600 physical therapists and obtain a nearly representative sample of this workgroup in Germany (n = 300).

#### First strategy of recruitment

##### Professional associations of physical therapists in Germany

All of the existing professional associations (n = 4) of physical therapists will be involved within this recruitment strategy (The professional physiotherapy associations in Germany are: (Bundesverband selbstständiger Physiotherapeuten (IFK) e. V.; Physiotherapieverband e. V. (VDB); Verband Physikalische Therapie (VPT); Deutscher Verband für Physiotherapie (ZVK) e. V.).

At first the national physiotherapeutic associations will be addressed and asked for general consent to participate. According to the specific organization of the individual professional associations, the drawing of the sample will be carried out by the national associations itself or subsamples will be drawn by the diverse national associations. Finally a random sample of 100 members per association should be included. A pseudonymized list of these subjects, including sex, age, and region, will be prepared by each professional association for the planned non-responder analysis. A total of 400 participants will be recruited in this way.

#### Second strategy of recruitment

##### Institution for Statutory Accident Insurance and Prevention for the Health and Welfare Services (BGW)

Each self-employed physical therapist in Germany is legally obliged to insure his or her employees within the BGW. Accident insurance for the self-employed themselves is voluntary. Therefore, the majority of physical therapists who work for private (non-governmental) institutions can be contacted through the BGW. This second strategy of recruitment will be two-staged. First, 100 institutions that employ physical therapists will be randomly selected from all physiotherapy practices insured by the BGW (n ≈ 46 500). Unfortunately, the number of physical therapists working in each establishment is not documented; therefore the exact number of subjects contacted will remain unclear. The selected institutions will be informed through the BGW about the aims and contents of the study and asked to participate. If an institution agrees, the enclosed reply has to be returned (by mail or fax) to the study coordination stating how many employees are working in their establishment. On the basis of these responses, the study coordination will send the appropriate number of requested questionnaires.

It is assumed that mostly supervisors of the addressed offices will be contacted by this second recruitment concept. Currently, it is difficult to assess whether this recruitment process will reach all (or at least an unbiased sample) of the contacted institutions’ salaried employees.

Only currently employed physical therapists aged between 18 and 65 years will be included. Masseurs, balneotherapists, respiratory therapists, occupational therapists and other therapeutically-related occupational groups, as well as, physical therapists which have not finished their training/academic studies will be excluded from the sample.

### External population-based comparison group - Inhabitants of a German city

A comparison group (n = 300) representing the general average risk of the outcomes of interest (musculoskeletal, dermal, infectious, mental complaints and diseases) will be included.

The recruitment of the comparison group will be carried out via the population registry office of Dresden. In concordance with the exposed group, 600 randomly selected inhabitants of Dresden will be contacted and asked to take the survey. Only those aged between 18 and 65 years (analogous to the group of physical therapists) meet the inclusion criteria.

Incentives and reminders (approximately four and eight weeks after initial contact) will be applied to increase the response of both groups.

## Data collection

### Standardized questionnaire-based survey

A self-administered standardized questionnaire manual will be given to both groups. To increase the response rate, all participants will be able to decide between an online or postal version of the survey. Additionally, all survey participants can participate in a drawing of online shopping vouchers.

The questionnaire manual has been compiled especially for this study. Existing validated and established measurement tools were used for the development of the manual. All of the measurement tools included and the contextual justification of each question are shown in Table 1. The Online-Version will be conducted using SoSci Survey (a professional software package for online surveys (https://www.soscisurvey.de/)).

**Table 1:** Exposures, outcomes and appropriate questionnaires included in the standardized survey

**Questions**	**Content**	**Reference(s)**
**A 1**	Job title	Self-formulated question
**A 2**	Terms of employment	Self-formulated question
**A 3-4**	Occupation (duration of occupation)	Nordic questionnaire [29]
**A 5-9**	Current work activity (working hours, non-working time/break, overtime, etc.)	Self-formulated question
**A 10-11**	Occupational history and job change for health reasons	Nordic Questionnaire [29]
**A 12**	Predominant workload at work	Copenhagen psychosocial questionnaire (COPSOQ) – German version [30]
**A 13-18**	Precise work activities (sector, work area, main activities, on-going training, etc.) - only physical therapists	Self-formulated question
**A 19**	Exposure assessment: musculoskeletal – only physical therapists	Risk factors for work-related musculoskeletal complaints by Campo et al., 2008 [14] – own translation
**A 20**	Exposure assessment: musculoskeletal (back)	Adapted question: EPILIFT [31]
**A 21**	Exposure assessment: musculoskeletal (in general)	Adapted question: EPILIFT [31]
**A 22-23**	Exposure assessment: musculoskeletal (strength and hand activity combined)	Hand activity level threshold limit values (HAL TLVs) [32]
**B 1-6**	Psychosocial demands and workload of the current occupation	Shorted version of Copenhagen psychosocial questionnaire (COPSOQ) – German version [30]
**B 7**	Assessment of effort reward imbalance	Effort-reward-imbalance questionnaire (ERI) Item version (short generic measure) [33]
**B 8**	Assessment of job (in-)security	ERI item version (short generic measure) [33]
**C 1**	Presenteeism	Own translation of Aronsson et al., 2000 [34]
**C 2**	Absenteeism	Self-formulated question
**D 1-4**	Musculoskeletal workload and complaints	Nordic questionnaire (German version) [29]
**E 1-2**	Individual risk factors for dermal conditions	Adapted question from chronic hand eczema registry on longterm patient management (CARPE) [35]
**E 2-8**	Dermal exposures and diseases	Nordic work-related skin questionnaire – German translation (linguistic translation, current preparation of publication) [36]
**F 1-2**	Exposure assessment: infectious	Self-formulated question
**F 3**	Contact or infection with reportable infectious diseases	Selection of relevant infectious diseases from the review by [37]
**G 1-12**	**Socio-demographic data**	
**G 1**	Sex	German demografic standards [38]
**G 2**	Marital status	German demografic standards [38]
**G 3-5**	Age, body height, body weight	Nordic questionnaire [29]
**G 6-7**	Graduation, occupational qualification	German demografic standards [38]
**G 8**	Hand-dominance	Adapted Nordic questionnaire [29]
**G 9**	Sport	Nordic questionnaire [29]; GEDA 2009 [39]
**G 10**	Smoking behaviour	Extra-short version to assess current smoking behaviour [40]
**G 11**	Previous illness (e.g. mental diseases etc.)	Self-formulated question

A follow-up is planned 3 to 5 years after this cross-sectional study (baseline) using the same manual.

### Clinical examination

To objectify the self-reported data of the health-related outcomes and to verify potential differential misclassifications, a random sample of the study participants will be given a clinical examination conducted by a physician and/or trained physical therapist. Based on the response of the standardized questionnaire-based survey, a sample size of approximately 100 subjects, with and without symptoms or diseases is planned and will comprise 50 physical therapists (n = 25 with and n = 25 without symptoms or diseases) and 50 subjects of the comparison group (n = 25 with and n = 25 without symptoms or diseases). The clinical examination will be conducted in accordance with the approach of the International Academy of Orthopedic Medicine (IAOM) [41]. In order to increase the response rate, all participants of the clinical examination will get a detailed evaluation of individual results as well as an individual counseling.

The examination will focus also primarily on musculoskeletal symptoms and diseases. Furthermore, dermal problems will be considered.

Musculoskeletal symptoms and diseases: First, an orthopedic examination [41] based on a general inspection/screening of the body and differentiated examinations of several body areas (spine, muscles/joints of the upper and lower extremities) will be conducted. In this context, the range of joint motion, muscular strength and any existing pain will be considered. All body parts will be subjected to the appropriate baseline examinations. Only upon the occurrence of irregularities or complaints special additional physical examination tests will be carried out.

Dermal symptoms/diseases will be assessed by means of a stepwise diagnostic procedure. Subjects with noticeable indications of hand and/or forearm eczema will be in cases of consent–examined with a specific diagnostic tool by a dermatologist. In this context the “Physician Global Assessment Score’’(PGA) [42] will be applied.

**Figure 1:**
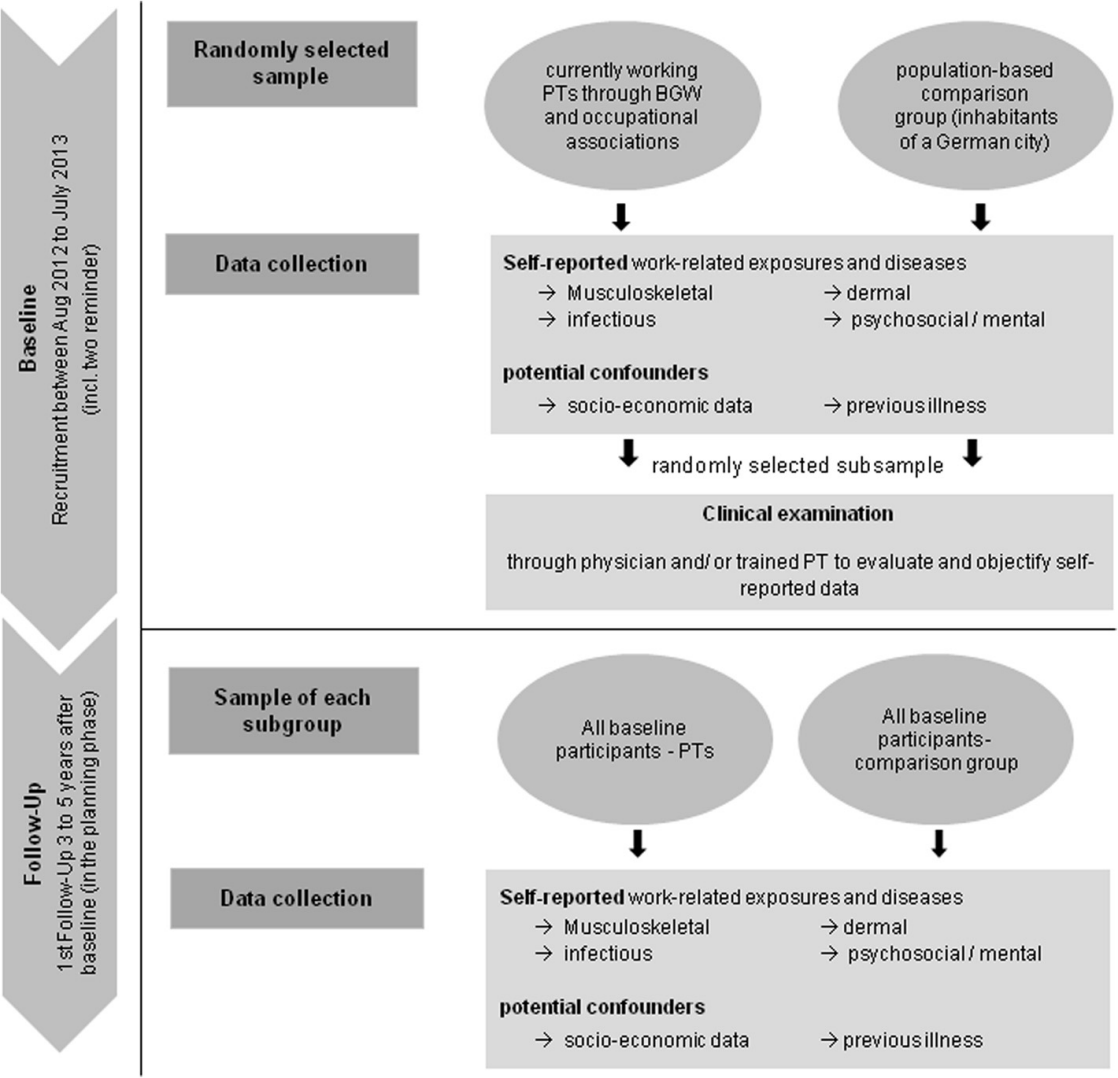
Recruitment strategies and data collection of the PTC study.

A summary of the recruitment process and data collection is given in Figure 1*.*

### Confounders

Besides the wide spectrum of the work-related exposures and complaints as well as diseases, potential confounders which could bias the association between exposure and outcome, will be assessed. Consequently, to diminish the influence of confounding, socio-demographic factors (age, sex), body mass index and lifestyle choices (smoking, sport activities) will be observed.

### Statistical analyses

The results will be evaluated descriptively and analytically. First, in a descriptive analysis, prevalence rates for musculoskeletal, dermal, infectious, and psychological complaints and diseases among physical therapists in Germany with defined work-related exposures will be reported. Second, prevalence ratios (PR) [43] will be applied to analyze the association between work as a physical therapist and specific diseases. Results yielding a p-value of <0.05 will be considered as statistically significant. All tests will be two-sided and be performed in IBM SPSS, version 19 and SAS (for concrete analyses see Table 2).

**Table 2:** Statistical analysis of the standardized questionnaire-based survey

**Prevalence**	
Descriptive analysis	▪ Musculoskeletal exposures as well as musculoskeletal complaints and diseases
	▪ Psychosocial exposures as well as mental complaints and diseases
	▪ Dermal complaints and diseases
	▪ Infectious diseases
**Prevalence ratios**	
Analytical analysis	▪ Musculoskeletal complaints among the group of physical therapists vs. comparison group (inhabitants of a German city) in relation to work area, and main work tasks in general as well as work-related musculoskeletal exposures in detail
	▪ Mental complaints among the group of physical therapists vs. comparison group (inhabitants of a German city) in relation to work-related psychosocial exposures as well as the actual form of employment
	▪ Dermal complaints among the group of physical therapists vs. comparison group (inhabitants of a German city) in relation to work area as well as work-related dermal exposures
	▪ Occurrence of infectious diseases among the group of physical therapists vs. comparison group (inhabitants of a German city) in relation to work area, field of specialization, as well as work-related infectious agents

Additionally, differences between the results of the clinical and the standardized questionnaire will also be tested. This will provide a means to gauge how well the questionnaire performs (with respect to musculoskeletal and dermal indications). Sensitivity, specificity, and accuracy of the Nordic Questionnaire (German version) [36] vs. clinical examination as well as self-reported skin diseases vs. PGA [42] will be calculated. Furthermore, results will be quantified between the included groups (physical therapists and inhabitants of a German city) regarding a possible differential misclassification.

### Quality control/Pretest

According to the German Guidelines and Recommendations to assure Good Epidemiologic Practice (GEP), an internal quality control is indispensable for each epidemiological study. This will be realized through cooperating partners (MD, UL, AN). Regular meetings and telephone conferences will be conducted.

Before finalization of the standardized manual, its comprehensiveness and acceptance was pre-tested with 20 subjects. In addition, the feasibility of the online survey was tested as part of the pretest.

A non-responder analysis is planned based on pseudonymized data to detect potential systematic socio-demographic differences between responders and non-responders.

### Ethic

This study was approved by the Ethic Committee of the University Hospital Dresden and the Saxony Commission of Data Protection. Furthermore, planning and execution of this study will also be conducted in accordance to the GEP [44] of the German Society for Epidemiology (German: Deutsche Gesellschaft für Epidemiologie).

The project implementation and the resulting publications will also follow the Rules of Good Scientific Practice of the German Research Foundation (German: Deutsche Forschungsgemeinschaft, DFG) [45].

## Discussion

The expected results of this study should provide initial evidence regarding main work-related exposures and diseases of physical therapists in Germany. Due to the lack of a registry of physical therapists in Germany, the representativeness of the included physiotherapists might be limited. Within the planning phase it remains unclear if the required power for work-related exposures and outcome for our prospective cohort study will be sufficient. For the prospective analysis, the following outcomes are taken into consideration: incident musculoskeletal complaints and diseases, recurrent diseases, and course of disease.

## Abbreviations

BGW: Institution for statutory accident insurance and prevention in the health and welfare services; (CARPE): Chronic hand eczema registry on longterm patient management; (COPSOQ): Copenhagen Psychosocial Questionnaire; DFG: Deutsche Forschungsgemeinschaft; (ERI): Effort-reward-imbalance questionnaire; GEP: Good epidemiologic practice; MRSA: Methicillin-resistant staphylococcus aureus; PGA: Physician global assessment score; PR: Prevalence ratios; PTC: Physical therapist cohort; SAS: Statistical analysis systems; SPSS: Statistical package for the social sciences

## Competing interests

The authors report no competing interests. The funder has no role in the analyses of the study.

## Authors’ contributions

MG: drafting/revising of the manuscript, study concept and design, study coordination. SD: drafting/revising of the manuscript, concretion of study design, design of the questionnaire manual. CK: revising of the manuscript, concretion of study design, design of the questionnaire manual, planning of clinical examinations. UL: external quality control, selection of items for the questionnaire. MD: external quality control, selection of items for the questionnaire. AN: external quality control. DG: external quality control, selection of items for the questionnaire. AS: study concept and design, study supervision. All authors approved and critically reviewed the final version of the manuscript.
